# The experience of mathematical beauty and its neural correlates

**DOI:** 10.3389/fnhum.2014.00068

**Published:** 2014-02-13

**Authors:** Semir Zeki, John Paul Romaya, Dionigi M. T. Benincasa, Michael F. Atiyah

**Affiliations:** ^1^Wellcome Laboratory of Neurobiology, University College LondonLondon, UK; ^2^Department of Physics, Imperial College LondonLondon, UK; ^3^School of Mathematics, University of EdinburghEdinburgh, UK

**Keywords:** mathematics, neuroesthetics, fMRI, beauty, mofc

## Abstract

Many have written of the experience of mathematical beauty as being comparable to that derived from the greatest art. This makes it interesting to learn whether the experience of beauty derived from such a highly intellectual and abstract source as mathematics correlates with activity in the same part of the emotional brain as that derived from more sensory, perceptually based, sources. To determine this, we used functional magnetic resonance imaging (fMRI) to image the activity in the brains of 15 mathematicians when they viewed mathematical formulae which they had individually rated as beautiful, indifferent or ugly. Results showed that the experience of mathematical beauty correlates parametrically with activity in the same part of the emotional brain, namely field A1 of the medial orbito-frontal cortex (mOFC), as the experience of beauty derived from other sources.

## Introduction

“Mathematics, rightly viewed, possesses not only truth, but supreme beauty”

Bertrand Russell, *Mysticism and Logic* (1919).

The beauty of mathematical formulations lies in abstracting, in simple equations, truths that have universal validity. Many—among them the mathematicians Bertrand Russell (1919) and Hermann Weyl (Dyson, 1956; Atiyah, 2002), the physicist Paul Dirac (1939) and the art critic Clive Bell (1914)—have written of the importance of beauty in mathematical formulations and have compared the experience of mathematical beauty to that derived from the greatest art (Atiyah, 1973). Their descriptions suggest that the experience of mathematical beauty has much in common with that derived from other sources, even though mathematical beauty has a much deeper intellectual source than visual or musical beauty, which are more “sensible” and perceptually based. Past brain imaging studies exploring the neurobiology of beauty have shown that the experience of visual (Kawabata and Zeki, 2004), musical (Blood et al., 1999; Ishizu and Zeki, 2011), and moral (Tsukiura and Cabeza, 2011) beauty all correlate with activity in a specific part of the emotional brain, field A1 of the medial orbito-frontal cortex, which probably includes segments of Brodmann Areas (BA) 10, 12 and 32 (see Ishizu and Zeki, 2011 for a review). Our hypothesis in this study was that the experience of beauty derived from so abstract an intellectual source as mathematics will correlate with activity in the same part of the emotional brain as that of beauty derived from other sources.

Plato (1929) thought that “nothing without understanding would ever be more beauteous than with understanding,” making mathematical beauty, for him, the highest form of beauty. The premium thus placed on the faculty of understanding when experiencing beauty creates both a problem and an opportunity for studying the neurobiology of beauty. Unlike our previous studies of the neurobiology of musical or visual beauty, in which participating subjects were neither experts nor trained in these domains, in the present study we had, of necessity, to recruit subjects with a fairly advanced knowledge of mathematics and a comprehension of the formulae that they viewed and rated. It is relatively easy to separate out the faculty of understanding from the experience of beauty in mathematics, but much more difficult to do so for the experience of visual or musical beauty; hence a study of the neurobiology of mathematical beauty carried with it the promise of addressing a broader issue with implications for future studies of the neurobiology of beauty, namely the extent to which the experience of beauty is bound to that of “understanding.”

## Materials and methods

Sixteen mathematicians (3 females, age range = 22–32 years, 1 left-handed) at postgraduate or postdoctoral level, all recruited from colleges in London, took part in the study. All gave written informed consent and the study was approved by the Ethics Committee of University College London. All had normal or corrected to normal vision. One subject was eliminated from the study after it transpired that he suffered from attention deficit hyperactivity disorder and had been on medication, although his exclusion did not affect the overall results. We also recruited 12 non-mathematicians who completed the questionnaires described below but were not scanned, for reasons explained below.

### Experimental procedure

To allow a direct comparison between this study and previous ones in which we explored brain activity that correlates with the experience of visual and musical beauty (Kawabata and Zeki, 2004; Ishizu and Zeki, 2011), we used similar experimental procedures to these previous studies. About 2–3 weeks before the scanning experiment, each subject was given 60 mathematical formulae (Data Sheet 1: EquationsForm.pdf) to study at leisure and rate on a scale of −5 (ugly) to +5 (beautiful) according to how beautiful they experienced them to be. Two weeks later, they participated in a brain scanning experiment, using functional magnetic resonance imaging (fMRI), during which they were asked to re-rate the same equations while viewing them in a Siemens scanner, on an abridged scale of ugly—neutral—beautiful. The pre-scan ratings were used to balance the sequence of stimuli for each subject to achieve an even distribution of preferred and non-preferred equations throughout the experiment. A few days after scanning, each subject received a questionnaire (Data Sheet 2: UnderstandingForm.pdf) asking them to (a) report their level of understanding of each equation on a numerical scale, from 0 (no understanding) to 3 (profound understanding) and (b) to report their subjective feelings (including emotional reaction) when viewing the equations. The data from these questionnaires (pre-scan beauty ratings, scan-time beauty ratings, and post-scan understanding ratings) is given in Data Sheet 3: BehavioralData.xls.

### Stimuli

Stimuli consisting of equations were generated using Cogent 2000 (http://www.vislab.ucl.ac.uk/Cogent2000) and displayed by an Epson EH-TW5910 LCD projector at a resolution of 1600 × 1200 with a refresh rate of 60 Hz. The display was back-projected onto a translucent screen (290 × 180 mm, 27.2° × 18.1° visual angle), which was viewed by subjects using an angled mirror.

### Scanning

Subjects viewed the formulae during four functional scanning sessions, with breaks between sessions which gave them an opportunity to take a rest if required and us to correct any anomalies, for example to correct rare omissions in rating a stimulus. Scans were acquired using a 3-T Siemens Magnetom Trio MRI scanner fitted with a 32-channel head volume coil (Siemens, Erlangen, Germany). A B0 fieldmap was acquired using a double-echo FLASH (GRE) sequence (duration 2′ 14″). An echo-planar imaging (EPI) sequence was applied for functional scans, measuring BOLD (Blood Oxygen Level Dependent) signals (echo time *TE* = 30 ms, *TR* = 68 ms, volume time = 3.264 s). Each brain image was acquired in an ascending sequence comprising 48 axial slices, each 2 mm thick, with an interstitial gap of 1 mm and a voxel resolution of 3 × 3 × 3 mm, covering nearly the whole brain. After functional scanning had been completed a T1 MDEFT (modified driven equilibrium fourier transform) anatomical scan was acquired in the saggital plane to obtain high resolution structural images (176 slices per volume, constant isotropic resolution 1 mm, *TE* = 2.48 ms, *TR* = 7.92 ms).

**Figure 1:**
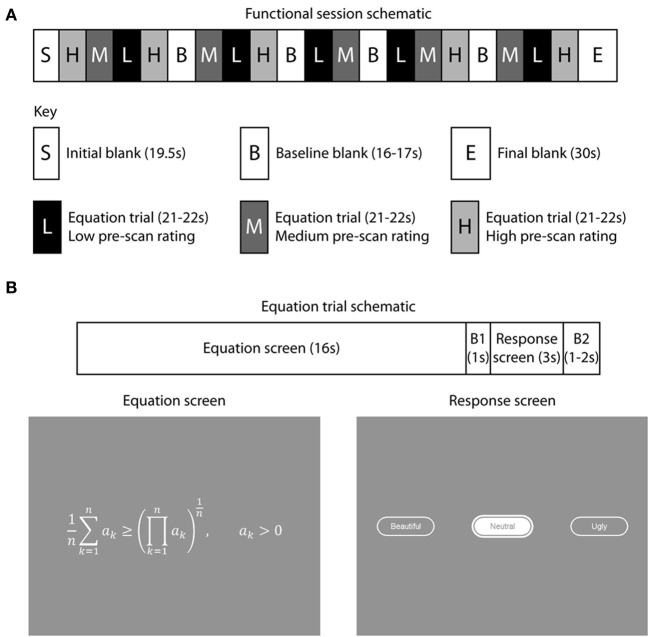
**Experimental paradigm and stimulus presentation**. There were four functional scanning sessions, with a break between sessions. **(A)** 15 equations were displayed during each session, with each of the 60 equations appearing only once over the four sessions. The pre-scan beauty ratings were used to sequence the equations for each subject so that 5 low-rated (L), 5 medium-rated (M), and 5 highly-rated equations (H) appeared in each session and their sequence within a session organized so that a low-rated equation was never followed by another low-rated equation (similarly for medium and highly-rated equations). During each session four blank periods, each varying in duration from 16 to 17 s, were inserted to collect baseline signal. Each session commenced with a 19.5 s blank period to allow T1 equilibration effects to subside and ended with a 30 s blank period, giving a total duration of about 7 min 20 s per functional session. **(B)** Each equation trial began with an equation displayed for 16 s, followed by a response period during which subjects indicated their scan-time beauty rating by pressing one of three keypad buttons. This started with a 1 s blank period (B1) followed by 3 s during which subjects pressed keypad buttons and their selection (either “Ugly,” “Neutral,” or “Beautiful”) was interactively displayed to them. The response was followed by an inter-trial interval which was randomly varied between 1 and 2 s during which a blank screen appeared (B2). Blank periods displayed a uniform mid-gray background.

Fifteen equations were displayed during each session (Figure 1A), so that each of the 60 equations appeared once over the four sessions. Each session started with a blank gray screen for 19.5 s, followed by 15 trials, each of 16 s, interspersed with four blanks, each of 16–17 s, to acquire baseline signal. A plain gray blank screen was used, of an equivalent overall brightness to the equation screens. Pre-scan beauty ratings were used to divide the 60 equations into three groups; 20 “low” rated, 20 “medium” rated, and 20 “high” rated equations. The sequence of equations viewed by each subject in the scanner was then organized so that 5 low-, 5 medium- and 5 highly-rated equations appeared in each session and the pseudo-randomized sequence was organized so that a low-rated equation was never followed by another low-rated equation and the same held for medium and highly rated equations. The session ended with a blank screen of duration 30 s.

Each trial (Figure 1B) began with an equation which was displayed for 16 s followed by a blank lasting 1 s. The response screen appeared for 3 s, during which the subject selected interactively a scan-time beauty rating (Beautiful, Neutral, or Ugly) for each equation by pressing keypad buttons. A second blank lasting 1–2 s ended each trial. Equations were all drawn in the same sized font in white (CIE 1931 XYZ: 755, 761, 637) and the same gray background was used throughout (CIE 1931 XYZ: 236, 228, 200). The overall screen brightness varied between 280 and 324 cd m^−2^; the width of equations varied from 4° to 24° visual angle and the height varied between 1° and 5°.

### Analysis

SPM8 (Statistical Parametric Mapping, Friston et al., 2006) was used to analyze the results, as in our previous studies (Zeki and Romaya, 2010; Ishizu and Zeki, 2011). At the single-subject level the understanding rating (0–3) and the scan-time beauty rating (coded as −1 for “Ugly,” 0 for “Neutral,” and 1 for “Beautiful”) for each equation were included as first and second parametric modulators, respectively, of a boxcar function which modeled the appearance of each equation [in fact, the beauty and understanding ratings correlated but imperfectly (see behavioral data below)]. There were fewer “Ugly” rated equations than “Neutral” or “Beautiful” (see behavioral data below). Indeed, in 2 of the 60 functional sessions there was no “Ugly” rating. This imbalance does not bias the estimation of, or inference about, the effects of beauty—it only reduces the efficiency with which the effects can be estimated (Friston et al., 2000). Happily, this reduction was not severe, because we were able to identify significant effects. As a result of SPM orthogonalization, the beauty rating parametric modulator can only capture variance that cannot be explained by the understanding rating, thus allowing us to distinguish activations that correlate with beauty alone. Contrast images for each of the 15 subjects for the parametric beauty rating and for All equations vs. Baseline were taken to a 2nd-level (random effects) analysis. We used a conjunction-null analysis (Nichols et al., 2005), to determine whether there was an overlap, within mOFC, in regions of parametric activation with beauty and the general de-activation produced by viewing mathematical formulae. In order to examine the activity of Ugly, Neutral, and Beautiful stimuli relative to baseline at locations identified in the parametric beauty analysis we also carried out a categorical analysis of the beauty rating alone, by coding contrasts for Ugly, Neutral, and Beautiful stimuli vs. Baseline for each subject at the first level and taking these to a 2nd level, random effects, analysis as before.

In a similar way, we undertook another parametric analysis with the scan-time beauty rating as the first parametric modulator and the understanding rating as the second one. This time the understanding modulator can only capture variance that cannot be explained by the beauty rating, thus allowing us to distinguish activations that are accounted for by understanding alone. Contrast images for the 15 subjects were taken to the second level, as before. To supplement this we also undertook a categorical analysis of the four understanding categories (0–3) in order to obtain parameter estimates for the four understanding categories at locations identified as significant in the parametric understanding analysis (see Figure 5B).

A categorical analysis differs from the corresponding parametric analysis in two respects: first, the parametric analysis looks for a relationship between BOLD signal and differences in the rated quantity (beauty or understanding) on an individual session basis, while a categorical analysis will average the BOLD signal for each category of the rated quantity over all sessions and subjects; second, when using two parametric modulators we can isolate beauty effects from understanding and vice versa by using orthogonalization, but this is not available in a categorical analysis. When we use orthogonalization of two parametric regressors to partition the variance in the BOLD signal into a “beauty only” and an “understanding only” component, there remains a common portion which cannot be directly attributed to either component.

For the main contrasts of interest, parametric, and categorical beauty, we report activation at cluster-level significance (**P_Clust-FWE_** < 0.05) with familywise error correction over the whole brain volume as reported by SPM8 based on random field theory (Friston et al., 1994). A statistical threshold of **P_unc._** < 0.001 and an extent threshold of 10 voxels was used to define clusters. There were also some activations which did not reach significance (clearly noted) which we nevertheless report since they suggest areas which may be contributing to the main activation. Whether they actually do will be left to future studies.

For other contrasts vs. baseline which are not of principal interest to this study we report activations that survive a peak voxel threshold of **P**_**FWE**_ < 0.05, with familywise error correction over the whole brain volume.

Co-ordinates in millimeters are given in Montreal Neurological Institute (MNI) space (Evans et al., 1993).

## Results

### Behavioral data

#### Beauty ratings

The formula most consistently rated as beautiful (average rating of 0.8667), both before and during the scans, was Leonhard Euler's identity

$$1+e^{i\pi }=0$$

which links 5 fundamental mathematical constants with three basic arithmetic operations, each occurring once; the one most consistently rated as ugly (average rating of −0.7333) was Srinivasa Ramanujan's infinite series for 1/π,

$$\frac{1}{\pi }=\frac{2\sqrt{2}}{9801}\sum_{k\;=\;0}^{\infty }\frac{\left(4k\right)!\left(1103+26390k\right)}{{\left(k!\right)}^{4}396^{4k}}$$

which expresses the reciprocal of π as an infinite sum.

Other highly rated equations included the Pythagorean identity, the identity between exponential and trigonometric functions derivable from Euler's formula for complex analysis, and the Cauchy-Riemann equations (Data Sheet 1: EquationsForm.pdf—Equations 2, 5, and 54). Formulae commonly rated as neutral included Euler's formula for polyhedral triangulation, the Gauss Bonnet theorem and a formulation of the Spectral theorem (Data Sheet 1: EquationsForm.pdf—Equations 3, 4, and 52). Low rated equations included Riemann's functional equation, the smallest number expressible as the sum of two cubes in two different ways, and an example of an exact sequence where the image of one morphism equals the kernel of the next (Data Sheet 1: EquationsForm.pdf—Equations 15, 45, and 59).

#### Pre-, post-, and scan-time ratings

In pre-scan beauty ratings, each subject rated each of the 60 equations according to beauty on a scale of −5 (Ugly) through 0 (Neutral) to +5 (Beautiful) while during scan time ratings, subjects rated each equation into the three categories of Ugly, Neutral or Beautiful.

***Post-scan understanding ratings.*** After scanning, subjects rated each equation according to their comprehension of the equation, from 0 (no comprehension whatsoever) to 3 (Profound understanding).

An excel file containing raw behavioral data is provided as Data Sheet 3: BehavioralData.xlsx., which gives the following eight tables:

Table 1: Pre-scan beauty ratings for each equation by subject
Table 2: Scan-time equation numbers by subject, session and trial
Table 3: Scan-time beauty ratings for each equation by subject
Table 4: Scan-time beauty ratings by subject, session, and trial
Table 5: Scan-time beauty ratings by subject—Session and experiment totals
Table 6: Post-scan understanding ratings by subject
Table 7: Post-scan understanding ratings by subject, session, and trial
Table 8: Post-scan understanding ratings by subject—Session and experiment totals.

**Figure 2:**
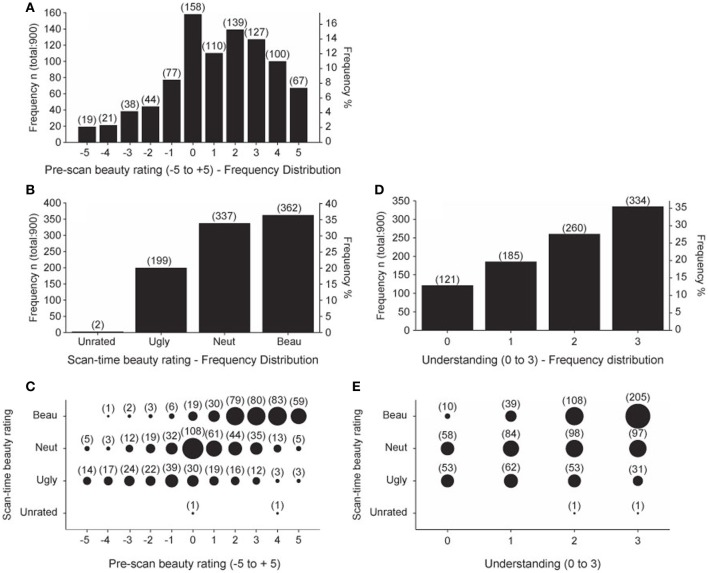
**Summary of Behavioral data**. Behavioral data scores summated over all 15 subjects. **(A)** Frequency distribution of pre-scan beauty ratings. **(B)** Frequency distribution of scan-time beauty ratings. **(C)** Pre-scan beauty ratings plotted against scan-time beauty ratings. **(D)** Frequency distribution of post-scan understanding ratings. **(E)** Post-scan understanding ratings plotted against scan-time beauty ratings. Numbers in brackets give the count for each group. Area of each circle is proportional to the count for that group.

The pre-scan beauty ratings were used to assemble the equations into three groups, one containing 20 low-rated, another 20 medium-rated, and a third 20 high-rated equations, individually for each subject. These three allocations were used to organize the sequence of equations viewed during each of the four scanning sessions so that each session contained 5 low-rated, 5 medium-rated, and 5 high-rated equations. Each subject then re-rated the equations during the scan as Ugly, Neutral, or Beautiful. In an ideal case, each subject would identify 5 Ugly, 5 Neutral, and 5 Beautiful equations in each session. In fact, this did not happen. Figure 2A shows the frequency distribution of pre-scan beauty ratings for all 15 subjects; it is positively skewed, indicating that more equations were rated as beautiful than ugly. This is reflected in the frequency distribution of the scan-time beauty ratings (Figure 2B) which, again, shows a bias for beautiful equations. Figure 2C shows the relationship between pre-scan and scan-time beauty ratings. There was a highly significant positive correlation (Pearson's *r* = 0.612 for 898 values, *p* < 0.001) but there were departures; for example, one equation received a pre-scan rating of −4 but was classed as Beautiful at scan-time and three equations with a pre-scan rating of 5 were subsequently classed as Ugly. These infrequent departures are not of great concern providing there was still a reasonable ratio of Ugly: Neutral: Beautiful scan-time designations for each session, which was the case. Ideally this ratio would always be 5:5:5 but, due to the predominance of Beautiful over Ugly scan-time ratings, we twice recorded 0:7:8 and 1:5:9 for particular sessions (see Table 5 in Data Sheet 3: BehavioralData.xlsx). Other sessions in general showed more equable ratios and, even with an extreme ratio such as 0:7:8, a relationship between Neutral and Beautiful equations could still be established.

The frequency distribution of post-scan understanding ratings is given in Figure 2D, which shows that more of the equations were well understood, as would be expected from a group of expert mathematicians. Figure 2E shows that there was a highly significant positive correlation (Pearson's *r* = 0.413 for 898 values, *p* < 0.001) between understanding and scan-time beauty ratings. In this case, departures from a fully correlated relationship allow us to separate out effects of beauty from those of understanding, so that, for example, in the well understood category (3) the ratio of Ugly: Neutral: Beautiful is 31:97:205. In order to analyze scanning data with regard to understanding ratings we would ideally have equal ratios of the four understanding ratios (0, 1, 2, and 3) in each scanning session. These ratios are recorded in Table 8 in Data Sheet 3: BehavioralData.xlsx. We occasionally find missing categories in some sessions (such as 0:2:4:9) but we could still establish a relationship when one category is missing in a particular session.

#### Post-scan questionnaires regarding subjective (emotional) experiences

Mathematical subjects were as well given four questions to answer, post-scan. One subject did not respond to this part of the questionnaire, leaving us with 14 subjects. To the question: “*When you consider a particularly beautiful equation, do you experience an emotional response?*,” 9 gave an unqualified “Yes,” 1 reported a “shiver of appreciation,” 1 reported being “a bit excited,” 1 reported “The same kind of response as when hearing a beautiful piece of music, or seeing a particularly appealing painting,” 1 reported that “the feeling is visceral” and 1 was “unsure” To the question: “*Do you derive pleasure, happiness or satisfaction from a beautiful equation*?” 14 subjects answered affirmatively; all 14 also gave a positive response to the question: “*Is there any mathematical equation which, in the past, you have found particularly beautiful and, if so, was it among the list of equations which we gave you*?” but some regretted that variations of the equations were not on the list [e.g., the Einstein field equations, related to equation 60 (contracted Bianchi identity), and Cauchy's integral formula for the special case where *n* = 1 (Equation 29)]; three regretted that the following equations were not on the list: the analytical solution of the Abel integral equation, Noether's theorem, the Euler-Lagrange and the Liouville, Navier-Stokes and Hamilton equations, Newton's Second Law (**F** = *m***a**), and the relativistic Dirac equation. Finally, variable answers were given to the question: “Do you experience a heightened state of consciousness when you contemplate a beautiful equation?” In summary, our subjects had an emotional experience when viewing equations which they had rated as beautiful (the “aesthetic emotion”), and which they also qualified as satisfying or pleasurable. They also showed a very sophisticated knowledge of mathematics, by specifying equations that they considered particularly beautiful (which they had known), as well as by the regret expressed at not finding, in our list, equations that they consider especially beautiful.

#### Non-mathematical subjects

We also tried to gauge the reaction of 12 non-mathematical subjects to viewing the same equations. This was, generally, an unsatisfactory exercise because many had had some, usually elementary, mathematical experience [up to GCSE (General Certificate of Secondary Education) level, commonly taken at ages 14–16]. Reflecting this, the majority indicated that they had no understanding of what the equations signified, rating them 0, although some gave positive beauty ratings to a minority of the equations. Overall, of the 720 equations distributed over 12 non-mathematical subjects, 645 (89.6%) were given a 0 rating (no understanding), 49 (6.8%) were given a rating of 1 (vague understanding) and the remainder were rated as 2 (good understanding) or 3 (profound comprehension). To the question “*When you consider a particularly beautiful equation, do you experience an emotional response?*,” the majority (9 out of 12) gave a negative response. Given this, we hypothesized that, when such non-mathematical subjects gave a positive beauty rating to the equations, they were doing so on a formal basis, that is to say on how attractive the form of the equations was to them. This hypothesis receives support from the contrast to reveal the parametric relationship between brain activity and understanding in mathematicians (see Results).

### Brain activations

#### Parametrically related activity in mOFC with beauty ratings, independently of understanding

**Figure 3:**
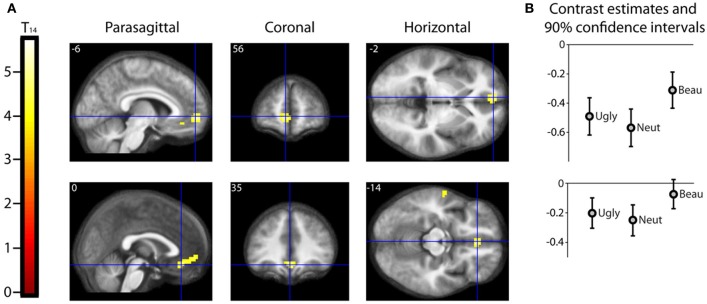
**Parametric “Activations” with Beauty. (A)** Second level parametric analysis derived from 15 subjects, to show parametric modulation by scan-time beauty rating (after orthogonalization to understanding rating). One-sample *t*-test (*df* = 14) thresholded at *P*_unc_ < 0.001 with an extent threshold of 10 voxels, revealed a cluster of 95 voxels in medial orbito-frontal cortex (mOFC) with hot-spots at (−6, 56, −2) and (0, 35, −14), significant at cluster level, with familywise error correction over the whole brain volume. The location and extent of the cluster is indicated by sections along the three principal axes through the two hot-spots, pinpointed with blue crosshairs and superimposed on an anatomical image which was averaged over all 15 subjects. (Note: “activation” in this case relates to a positive parametric relationship; i.e., an increase in activity with an increase in scan-time beauty rating. Overall, as can be seen in **(B)**, these locations were deactivated relative to baseline, i.e., there was a relative activation in a deactivated region. **(B)** A separate categorical analysis, based on scan-time beauty ratings alone, was used to generate contrast estimates for the three categories Ugly, Neutral, and Beautiful vs. Baseline at each of the locations in **(A)**.

Results should that activity that was parametrically related to the declared intensity of the experience of mathematical beauty was confined to field A1 of mOFC (Ishizu and Zeki, 2011), where there was a significant difference in the BOLD signal when viewing equations rated as beautiful on the one hand and as neutral and ugly on the other (Figure 3). Even though our subjects were experts, with an understanding of the truths that the equations depict, we had nevertheless asked them to rate, post-scan, how well they had understood the formulae, on a scale of 0 (no comprehension) to 3 (profound understanding) (Data Sheet 2: UnderstandingForm.pdf), as a way of dissociating understanding from the experience of beauty. There was a good but imperfect correlation between their understanding and the beauty ratings given, in that some formulae that had been understood were not rated as beautiful (Pearson's correlation coefficient ranging from −0.0362 to 0.6826). As described above, separate parametric modulators were used for understanding and beauty ratings in the SPM analysis. This allowed us to model both understanding and beauty effects and examine responses to one that could not be explained by the other, thus separating out the two faculties in neural terms. Hence, crucially, the parametrically related activity in mOFC (Figure 3) was specifically driven by beauty ratings, after accounting for the effects of understanding.

The mOFC was the only brain region that showed a BOLD signal that was parametrically related to beauty ratings, significant at cluster level (see Table 1A and Figure 3). Previous studies have nevertheless shown a number of areas that are active when subjects undertake mathematical tasks (see Arsalidou and Taylor, 2011 for a meta-analysis) and we observed some activity (which did not reach significance) in three regions which previous studies of mathematical cognition had reported to be active (see Table 1B). One of these is located in the left angular gyrus, one in the middle temporal gyrus and one in the caudate nucleus. Although they did not attain significance, we nevertheless document them here and leave it to future studies to ascertain their possible role in the experience of mathematical beauty.

**Table 1:** **Activation for the contrast *parametric beauty***.

**Location**	***x***	***y***	***z***	***k_E_***	***P*_Clust-FEW_**	***T*_14_**	***P*_FWE_**
**(A) ACTIVATIONS—*PARAMETRIC BEAUTY*—SIGNIFICANT AT CLUSTER LEVEL**							
mOFC	**−6**	**56**	**−2**	**95**	**0.047**	**5.14**	**0.619**
mOFC	0	35	−14			4.90	0.733
**(B) ACTIVATIONS—*PARAMETRIC BEAUTY*—NOT REACHING**							
**CLUSTER LEVEL SIGNIFICANCE**							
R caudate	12	8	19	17	0.703	5.72	0.369
L angular gyrus	−36	−55	22	28	0.489	4.96	0.705
L middle temporal gyrus	−63	−4	−17	16	0.725	4.48	0.895

SPM 2nd level analysis (15 subjects). One sample t-test (df = 14). (A) Cluster level significant activation with familywise error correction over the whole brain volume (**P**_**Clust-FWE**_ < 0.05, background threshold **P**_**unc**._ < 0.001, extent threshold 10 voxels). Co-ordinates of the two hotspots in the cluster are given in MNI space. (B) In addition to the significant cluster activation shown in Figure 3 and tabulated above in (A) there were some clusters which failed to reach significance but which are listed here as possible contributors to the main cluster of activation. For completeness we also give the peak significance at each location, **P**_**FWE**_, with familywise error correction over the whole brain volume. The peak activation in each cluster is shown in bold with up to two other peaks in the same cluster listed below.

#### Beauty—categorical analyses

A categorical analysis of Beauty ratings vs. Baseline is less sophisticated than a parametric analysis in two respects, for reasons given in the methods section. Nevertheless, we thought it useful to employ such an analysis to examine the parameter estimates for Ugly, Neutral and Beautiful vs. Baseline at the locations in mOFC identified as significant in the parametric study. Figure 3B shows the parameter estimates for the three beauty categories vs. baseline at the two locations in mOFC identified in the parametric analysis: at (−6, 56, −2) and (0, 35, −14). It is evident that, at both, overall activity within this area of deactivation was greater for Beautiful than for Neutral or Ugly stimuli. In neither case is a linear relationship particularly evident, probably due to the inferior sensitivity of the categorical analysis, for the reasons given above.

Table 2 tabulates the categorical contrast Beauty > Neutral. There are cluster-level significant activations in mOFC, the left angular gyrus and the left superior temporal sulcus. The relative activation within mOFC occurs within a region of de-activation relative to baseline (see section Cortical de-activations when viewing mathematical formulae). As with the previous study of Kawabata and Zeki (2004), parameter estimates show that it is a change in relative activity within a de-activated mOFC that correlates with the experience of mathematical beauty. Data Sheet 4: BeauNeutUglyBase.pdf tabulates activations and deactivations of the Beautiful, Neutral, and Ugly categories relative to baseline.

**Table 2:** **Activations for the categorical contrast *beautiful > neutral***.

**Location**	***x***	***y***	***z***	***k_**E**_***	***P*_Clust-FWE_**	***T*_14_**	***P*_FWE_**
**ACTIVATIONS—*BEAUTIFUL > NEUTRAL***							
L angular gyrus	**−48**	**−67**	**22**	**143**	**0.015**	**6.32**	**0.180**
mOFC	**−12**	**53**	**4**	**285**	<**0.001**	**5.52**	**0.416**
mOFC	−9	50	−5			5.16	0.576
mOFC	−9	38	−5			4.70	0.790
L superior temporal gyrus	**−12**	**29**	**61**	**122**	**0.026**	**4.98**	**0.633**
L superior temporal gyrus	−3	17	67			4.65	0.809
L superior temporal gyrus	−18	38	46			4.52	0.858

*SPM 2nd level analysis (15 subjects). One sample t-test (df = 14). Cluster level significant activations corrected for familywise error over the whole brain volume (**P**_**Clust-FWE**_ < 0.05, background threshold **P**_**unc**._ < 0.001, extent threshold 10 voxels). Co-ordinates of hotspots within each cluster are given in MNI space. For completeness we also give the peak significance at each location, **P**_**FWE**_, with familywise error correction over the whole brain volume. The peak activation in each cluster is shown in bold with up to two other peaks in the same cluster listed below*.

#### Activity unrelated to beauty ratings

While the experience of mathematical beauty correlated parametrically with activity in mOFC, the contrast All equations > Baseline showed that many sites were generally active when subjects viewed the equations (Table 3). These included sites implicated in a variety of relatively simple arithmetic calculations and problem solving (Dehaene et al., 1999; Fias et al., 2003; Anderson et al., 2011; Arsalidou and Taylor, 2011; Wintermute et al., 2012 for a meta-analysis), and symbol processing (Price and Ansari, 2011) as well as activity in three sites in the cerebellum, generally ignored in past studies of the mathematical brain: one of these cerebellar sites, located in Crus I, may be involved in working memory (Stoodley, 2012), another one, also located in Crus I, in grouping according to numbers (Zeki and Stutters, 2013) and the processing of abstract information (Balsters and Ramnani, 2008) while the third one, located in the para-flocculus, is involved in smooth pursuit eye movements (Ilg and Their, 2008). That these areas should have been active when subjects view more complex formulae suggests that they are also recruited in tasks that go beyond relatively simple arithmetic calculations and involve more complex mathematical formulations.

**Table 3:** **Activations and de-activations for the contrast *all equations vs. baseline***.

**Location**	***x***	***y***	***z***	***k*_E_**	***T*_14_**	***P*_FWE_**
**ACTIVATIONS—*ALL EQUATIONS > BASELINE***						
L fusiform gyrus	**−33**	**−82**	**−8**	**188**	**15.20**	<**0.001**
L fusiform gyrus	−30	−91	−5		14.96	<0.001
R fusiform gyrus	**42**	**−79**	**−11**	**377**	**14.98**	<**0.001**
R cerebellum Crus I	48	−70	−29		13.02	<0.001
R fusiform gyrus	39	−58	−11		10.58	0.001
L inferior temporal gyrus	**−48**	**50**	**4**	**77**	**13.40**	<**0.001**
L inferior temporal gyrus	**−48**	**−61**	**−11**	**79**	**11.80**	<**0.001**
L intraparietal sulcus	**−30**	**−67**	**46**	**88**	**10.29**	**0.002**
L lingual/fusiform gyrus	−30	−49	49		9.53	0.005
R cerebellum lobule VIII/crus I	**6**	**−79**	**−29**	**45**	**10.10**	0.002
R cerebellum dorsal paraflocculus	**30**	**−70**	**−50**	**13**	**10.04**	**0.003**
L inferior frontal gyrus	**−48**	**14**	**25**	**24**	**9.35**	**0.006**
R intraparietal sulcus	**30**	**−58**	**43**	**19**	**8.80**	**0.013**
**DE-ACTIVATIONS—*ALL EQUATIONS < BASELINE***						
L precuneus	**−12**	**−46**	**43**	**1188**	**18.22**	<**0.001**
R precuneus	15	−40	43		16.63	<0.001
L precuneus	−15	−40	52		14.73	<0.001
R fusifom gyrus	**60**	**−52**	**16**	**179**	**15.39**	<**0.001**
Fusiform gyrus	63	−34	16		10.87	<0.001
Fusiform gyrus	63	−22	16		8.25	0.027
R medial orbito-frontal cortex	**3**	**32**	**−5**	**459**	**14.91**	<**0.001**
L medial orbito-frontal cortex	−6	23	−8		14.25	<0.001
R medial orbito-frontal cortex	12	53	−2		13.37	<0.001
R superior temporal gyrus	**57**	**−16**	**−5**	**256**	**14.53**	<**0.001**
R superior temporal gyrus	63	2	−23		14.39	<0.001
R superior temporal gyrus	45	−13	−8		11.05	<0.001
L lingual gyrus	**−12**	**−70**	**−2**	**21**	**11.81**	<**0.001**
L middle temporal gyrus	**−60**	**−4**	**−17**	**44**	**11.75**	<**0.001**
L superior medial gyrus (anterior paracingulate)	**0**	**53**	**22**	**33**	**10.76**	**0.001**
L middle frontal gyrus	**−24**	**26**	**40**	**16**	**10.46**	**0.002**
R superior frontal gyrus (anterior paracingulate)	**18**	**50**	**19**	**12**	**10.38**	**0.002**
L lingual gyrus	**−24**	**−46**	**−2**	**11**	**9.41**	**0.006**
R cuneus	**12**	**−88**	**16**	**11**	**8.98**	**0.010**

*SPM 2nd level analysis (15 subjects). One sample t-test (df = 14). All loci, in Montreal Neurological Institute (MNI) space, are significant, thresholded at **P**_**FWE**_ < 0.05 with familywise error correction over the whole brain volume and with an extent threshold of 10 voxels. The peak activation in each cluster is shown in bold with the cluster size in voxels (**k**_**E**_) with up to two other peaks in the same cluster listed below*.

#### Cortical de-activations when viewing mathematical formulae

**Figure 4:**
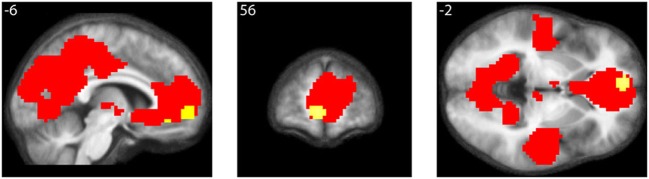
**Conjunction of activations with beauty rating and de-activations with equations**. Conjunction-Null of two contrasts, “Parametric beauty rating” and “De-activations with Equations,” both thresholded at P_unc_ < 0.001. De-activations are shown in red, overlapping the area revealed by the parametric rating, shown in yellow. Numerals refer to MNI co-ordinates.

As well, the contrast All equations < Baseline revealed widespread cortical de-activations (Table 3), many in areas not specifically related to mathematical tasks or aesthetic ratings; their distribution corresponds closely to areas active in the resting state and de-activated during complex cognitive tasks (Shulman et al., 1997; Binder et al., 1999), including arithmetic ones (Feng et al., 2007). The most interesting of these is in mOFC. A Conjunction-Null analysis (Nichols et al., 2005) of the contrasts “Parametric beauty rating” and “De-activations with Equations,” both thresholded at *P*_unc_ < 0.001, showed that this de-activation overlaps the mOFC activation which correlates parametrically with the experience of mathematical beauty (Figure 4); unlike other areas of de-activation, the mOFC de-activation has been posited to be related to task-unrelated conceptual processing (Shulman et al., 1997). The overlap between the activation and de-activation suggests that there may be separate compartments or sub-systems within field A1 of mOFC whose activities correlate with general cognitive tasks on the one hand and the more specific experience of beauty on the other.

#### Parametric “de-activations” with understanding ratings independent of beauty

**Figure 5:**
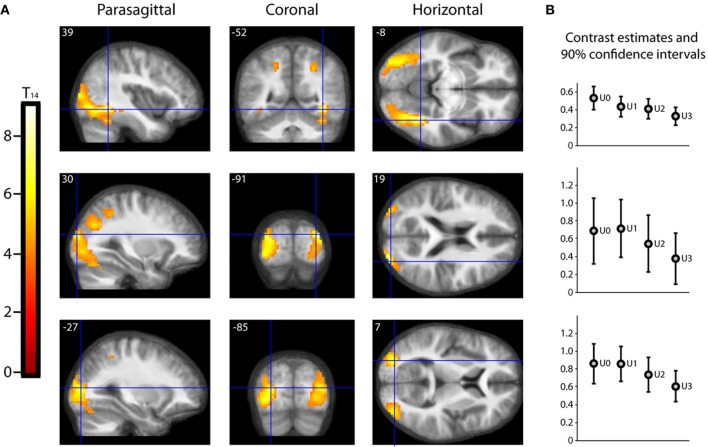
**Parametric “De-activations” with Understanding. (A)** Second level parametric analysis derived from 15 subjects, to show parametric modulation by understanding rating (after orthogonalization to scan-time beauty rating). One-sample *t*-test (*df* = 14) thresholded at *P*_unc_ < 0.001 with an extent threshold of 10 voxels, revealed two peak “de-activations” significant (**P**_**FWE**_ < **0.05**) with familywise error correction over the whole brain volume, at (39, −52, −8) (**T**_**14**_ = 9.01, **P**_**FWE**_ = 0.010) and at (30, −91, 19) (**T**_**14**_ = 8.05, **P**_**FWE**_ = 0.032). A third peak at (−27, −85, 7) (**T**_**14**_ = 7.65, **P**_**FWE**_ = 0.050) was just above threshold. The location of each peak is indicated by sections along the three principal axes, pinpointed with blue crosshairs and superimposed on an anatomical image which was averaged over all 15 subjects. (Note: A “de-activation” in this case relates to a negative parametric relationship; i.e., activity decreased as understanding rating increased. Overall, as can be seen in **(B)**, these locations were significantly active above baseline). **(B)** A separate categorical analysis based on understanding ratings alone was used to generate contrast estimates for the four understanding categories U0, U1, U2, and U3 vs. baseline at each of the peak locations in **(A)**.

As described in the methods, we undertook a second parametric analysis, with Beauty rating and Understanding rating as first and second parametric modulators, respectively, to isolate activations due to understanding alone. The result, shown in Figure 5, is that a large extent of the occipital visual cortex, comprising many of its subdivisions, was less active for well-understood equations (or, put another way, more active for less understood equations). The significance of this is discussed below (under Beauty and Understanding).

## Discussion

Art and mathematics are, to most, at polar opposites; the former has a more “sensible” source and is accessible to many while the latter has a high cognitive, intellectual, source and is accessible to few. Yet both can provoke the aesthetic emotion and arouse an experience of beauty, although neither all great art nor all great mathematical formulations do so. The experience of mathematical beauty, considered by Plato (1961a,b) to constitute the highest form of beauty, since it is derived from the intellect alone and is concerned with eternal and immutable truths, is also one of the most abstract emotional experiences. In spite of its abstract nature, there was, for Clive Bell (1914), a strong relation between mathematical and artistic beauty because the mathematician feels an emotion for his speculations which “springs… from the heart of an abstract science. I wonder, sometimes, whether the appreciators of art and of mathematical solutions are not even more closely allied,” while for Bertrand Russell (1919) “The true spirit of delight, the exaltation, the sense of being more than Man, which is the touchstone of the highest excellence, is to be found in mathematics as surely as poetry.” Given this, we hypothesized that it would be likely that the experience of beauty derived from mathematics would correlate with activity in the same part of the emotional brain as that derived from other, more sensible and perceptually based, sources. Although we approached the experiment with diffidence, given the profoundly different sources for these different experiences, we were not surprised to find, because of similarities in the experience of beauty provoked by the different sources alluded to above, that the experience of mathematical beauty correlates with activity in the same brain area(s), principally field A1 of mOFC, that are active during the experience of visual, musical, and moral beauty. That the activity there is parametrically related to the declared intensity of the experience of beauty, whatever its source, answers affirmatively a critical question in the philosophy of aesthetics, namely whether aesthetic experiences can be quantified (Gordon, 2005).

Mathematical and artistic beauty have been written of in the same breath by mathematicians and humanists alike, as arousing the “aesthetic emotion.” This implies that there is a common and abstract nature to the experience of beauty derived from very different sources. Viewed in that light, the activity in a common area of the emotional brain that correlates with the experience of beauty derived from different sources merely mirrors neurobiologically the same powerful and emotional experience of beauty that mathematicians and artists alike have spoken of.

### mOFC and the experience of beauty, preference, pleasure and reward

The mOFC is active in a variety of conditions, of which experiences relating to pleasure, reward and hedonic states are the most interesting in our context. The relationship of the experience of beauty to that of pleasure and reward has been commonly discussed in the philosophy of aesthetics, without a clear conclusion (Gordon, 2005). This is perhaps not surprising, because the three merge into one another, without clear boundaries between them; neurologically, activity in mOFC correlates with all three experiences thus reflecting, perhaps, the difficulty of separating these experiences subjectively. The imperfect distinction between the three is also reflected in the positive post-scan answers given by the mathematical subjects to the question whether they experienced pleasure, satisfaction or happiness when viewing equations that they had rated as beautiful. Whether one can ever experience beauty without at the same time experiencing a sense of pleasure and/or reward is doubtful. The converse is not true, in that something can be experienced as being pleasurable, rewarding, or preferred without it being also experienced as beautiful or arousing the “aesthetic emotion.” Neurobiologically, the issue resolves itself around the question of whether activity in the same or different parts of the mOFC correlates with these different experiences. The mOFC is a relatively large expanse of cortex with several cytocrachitectonic subdivisions, including BA 10, 11, 12, and 32 (see Kringelbach, 2005 for a review) as well as BA 24 (which may more properly be considered as part of the rostral anterior cingulate cortex) and BA 25. Brodmann maps are useful general anatomical guides but do not delimit functional areas and major functional subidvisions can be found within single cytoarchitectonic fields such as, for example, BA 18 (see Zeki, 1979). It is possible that, as with lateral orbito-frontal cortex, future studies may subdivide mOFC into further subdivisions based on resting-state connectivity or other criteria (Kahnt et al., 2012). Hence any reference to Brodmann maps is no more than a rough guide and must be tentative. Our delineation (Ishizu and Zeki, 2011) of a field within mOFC, field A1, has the virtue of delimiting a specific cortical zone with relatively precise co-ordinates and dimensions, whose activity correlates with the “aesthetic emotion” and to which other functional parcellations, both past and future, can be referred with relative precision. Field A1 of mOFC has MNI co-ordinates of (−3, 41, −8) and a diameter of between 15 and 17 mm (see Ishizu and Zeki, 2011). This falls in the middle of the active region of mOFC in the present study, and hence must be located within what we defined as field A1. In fact, if one were to take the average of the two hot spots in the mOFC cluster of the parametric beauty contrast of this study (namely −3, 45, −8), it will be found to be located 4 mm from the center of the field A1 at (−3, 41, −8). A survey of the published evidence shows that functional parcellations cannot be restricted to single cytoarchitectonic subdivisions comprising the medial orbital wall of the frontal cortex. Not all rewarding and pleasurable experiences activate field A1 of mOFC, which occupies mainly BA 32 but probably extends to BA 12 inferiorly and BA 25 anteriorly. While preference for drinks (McClure et al., 2004), abstract and predictive rewards and kinetic patterns do (O'Doherty et al., 2001; Gottfried et al., 2003; Zeki and Stutters, 2011), the hedonic experience of food appears to correlate with a more lateral part of mOFC than field A1 of mOFC (Kringelbach et al., 2003) while the experience of erotic pleasure appears to correlate with a region dorsal to it (Sescousse et al., 2010) (see also Berridge and Kringelbach, 2013) for a review. These are approximations that do not allow more definitive conclusions at present, even with meta-analyses such as the ones provided by Peters and Büchel (2010) or Kühn and Gallinat (2012). The latter help locate the general brain regions active with specific experiences but are not presently capable of pinpointing whether the identical regions are active, which would require far more detailed conjunction analyses in studies employing several different categories of hedonic experience. This is even true of the experience of visual and musical beauty. Though a conjunction analysis reveals that there is a common part of A1 of mOFC that correlates with both experiences (Ishizu and Zeki, 2011), the overlap is not perfect and it is possible that different subdivisions, or perhaps different groupings of cells within the same general subdivision, are recruited during different experiences (see Figure 1 of Ishizu and Zeki, 2011). The same is true of the present results in relation to previous activations of mOFC that correlate with the experience of visual and musical beauty. Very close correspondence in the active regions for all three experiences does not preclude that there may yet be different distributions or subdivisions within field A1 of mOFC. It is also useful to emphasize here, as we have done in the past, that the localization of activity in mOFC that correlates with the experience of mathematical beauty does not imply that this area alone is responsible for, or underlies, the experience. As we have shown here and elsewhere, the viewing of mathematical equations also results in activity in cortical areas that are distinct from the areas engaged when viewing paintings which, in turn, differ from those active when listening to musical excerpts. The common factor is activity in mOFC when beauty in these domains is experienced, but mOFC cannot act alone; rather it is active in concert with other areas which, we suppose, collectively correlate with the experience of beauty derived from different sources.

### Beauty and understanding

Perhaps one of the most awkward, and at the same time challenging, aspects of this work was trying to separate out beauty and understanding. Because the correlation between the two, though significant, was also imperfect, we were able to do so for mathematicians. This of course leaves the question of whether non-mathematicians, with no understanding whatsoever of the equations, would also find the equations beautiful. Ideally, one would want to have subjects who are mathematically totally illiterate, a search that proved difficult. We relied, instead, on a different approach. Most of our “non-mathematician” subjects had a very imperfect understanding of the equations, even though they had rated some of them as beautiful; we supposed that they did so on the basis of the formal qualities of the equations—the forms displayed, their symmetrical distribution, etc. We surmised that we could demonstrate this indirectly, by showing that less well understood equations in our mathematical subjects will, when viewed, lead to more intense activity in the visual areas. This is what we found (see Figure 5), and it implies that some combinations of form are more aesthetically pleasing than others, even if they are not “understood” cognitively (see also quote from Dirac, given below). What these formal qualities may be requires a separate and detailed study of the beauty of forms, well beyond the scope of this study. But a parallel may be found in our earlier study, which shows that there are some configurations of kinetic stimuli which activate the motion areas of the visual brain more intensely (Zeki and Stutters, 2011).

### Implications for future work

The experience of beauty derived from mathematical formulations represents the most extreme case of the experience of beauty that is dependent on learning and culture. The fact that the experience of mathematical beauty, like the experience of musical and visual beauty, correlates with activity in A1 of mOFC suggests that there is, neurobiologically, an abstract quality to beauty that is independent of culture and learning. But that there was an imperfect correlation between understanding and the experience of beauty and that activity in the mOFC cannot be accounted for by understanding but by the experience of beauty alone, raises issues of profound interest for the future. It leads to the capital question of whether beauty, even in so abstract an area as mathematics, is a pointer to what is true in nature, both within our nature and in the world in which we have evolved. Paul Dirac (1939) put it like this: “There is no logical reason why the (method of mathematical reasoning should make progress in the study of natural phenomena) but one has found in practice that it does work and meets with reasonable success. This must be ascribed to some mathematical quality in Nature, a quality which the casual observer of Nature would not suspect, but which nevertheless plays an important role in Nature's scheme… What makes the theory of relativity so acceptable to physicists in spite of its going against the principle of simplicity is its great mathematical beauty. This is a quality which cannot be defined, any more than beauty in art can be defined, but which people who study mathematics usually have no difficulty in appreciating. The theory of relativity introduced mathematical beauty to an unprecedented extent into the description of Nature… We now see that we have to change the principle of simplicity into a principle of mathematical beauty. The research worker, in his efforts to express the fundamental laws of Nature in mathematical form, should strive mainly for mathematical beauty. He should still take simplicity into consideration in a subordinate way to beauty. It often happens that the requirements of simplicity and of beauty are the same, but where they clash the latter must take precedence” (ellipses added). In similar vein, Hermann Weyl is recorded as having said, “My work always tried to unite the true with the beautiful; but when I had to choose one or the other, I usually chose the beautiful” (Dyson, 1956). Relevant here is the story of Weyl's mathematical formulations, which tried to reconcile electromagnetism with relativity. Rejected at first (by Einstein) because it was thought to conflict with experimental evidence, it came subsequently to be accepted but only after the advent of quantum mechanics, which led to a new interpretation of Weyl's equations. Hence the perceived beauty of his mathematical formulations ultimately predicted truths even before the full facts were known.

If the experience of mathematical beauty is not strictly related to understanding (of the equations), what can the source of mathematical beauty be? That is perhaps more difficult to account for in mathematics than in visual art or music. Whereas the source for the latter can be accounted for, at least theoretically, by preferred harmonies in nature or preferred distribution of forms or colors (see Bell, 1914; Zeki and Stutters, 2011; Zeki, 2013), it is more difficult to make such a correspondence in mathematics. The Platonic tradition would emphasize that mathematical formulations are experienced as beautiful because they give insights into the fundamental structure of the universe (see Breitenbach, 2013). For Immanuel Kant, by contrast, the aesthetic experience is as well grounded in our own nature because, for him, “Aesthetic judgments may thus be regarded as expressions of our feeling that something makes sense to us” (Breitenbach, 2013). We believe that what “makes sense” to us is grounded in the workings of our brain, which has evolved within our physical environment. Dirac (1939) wrote: “the mathematician plays a game in which he himself invents the rules while the physicist plays a game in which the rules are provided by Nature, but as time goes on it becomes increasingly evident that the rules which the mathematician finds interesting are the same as those which Nature has chosen” and therefore that in the choice of new branches of mathematics, “One should be influenced very much… by considerations of mathematical beauty” (ellipsis added). Hence the work we report here, as well as our previous work, highlights further the extent to which even future mathematical formulations may, by being based on beauty, reveal something about our brain on the one hand, and about the extent to which our brain organization reveals something about our universe on the other.

### Conflict of interest statement

The authors declare that the research was conducted in the absence of any commercial or financial relationships that could be construed as a potential conflict of interest.

## Supplementary material

We include four datasheets containing experimental details which are not essential for general understanding of the article:

Data Sheet 1 EquationsForm.pdf—The equation beauty-rating questionnaire.

Data Sheet 2 UnderstandingForm.pdf—The equation understanding questionnaire.

Data Sheet 3 BehavioralData.xlsx—Tables of behavioral data.

Data Sheet 4 BeauNeutUglyBase.pdf—Tables of categorical beauty activations vs. baseline.
