# Supplementary material for: The experience of mathematical beauty and its neural correlates
Source: Front Hum Neurosci. 2014 Feb 13;8:68. doi: 10.3389/fnhum.2014.00068 (PMC3923150; doi:10.3389/fnhum.2014.00068)
Supplement: Data Sheet 1 [file DataSheet1.PDF]

The Beauty of Mathematics

Equations

Please study the following equations, which each have a short description, but which are in no particular order. If you want to research any of them further, please go ahead. You should rate the beauty of each equation on a scale from -5 (ugly), through zero (neutral) up to +5 (beautiful).

Office use only			
Subject ID		Date returned	

#	Equation	Description	Rating
1	$1 + e^{i\pi} = 0$	Euler's identity links 5 fundamental mathematical constants with three basic arithmetic operations each occurring once.	
2	$\cos^2\theta + sin^2\theta = 1$	The Pythagorean identity, which states that for any angle, the square of the sine plus the square of the cosine is 1.	
3	$V - E + F = 2$	Euler's formula for triangulation of a polyhedron, where V is the number of vertices, E edges and F faces.	
4	$\int_M KdA + \int_{\partial M} k_g ds = 2\pi\chi(M)$	The Gauss-Bonnet theorem connects the geometry of surfaces (curvature) to their topology (Euler characteristic).	
5	$e^{ix} = \cos x + \, i\sin x$	Identity between exponential and trigonometric functions derivable from Euler's formula for complex analysis.	
6	$\int_{-\infty}^{\infty} e^{-x^2}\, dx = \sqrt{\pi}$	Definite Gaussian integral - ubiquitous in mathematical physics.	
7	$\frac{1}{\zeta(s)} = \sum_{n=1}^{\infty} \frac{\mu(n)}{n^s}, \quad s \in \mathbb{C}, \quad \mathrm{Re}(s) > 1$	The reciprocal of the zeta function can be expressed as a Dirichlet series over the Möbius function $\mu(n)$	
8	$exp(X) = \sum_{n=0}^{\infty} \frac{X^n}{n!}$	Series expansion for the exponential function.	
9	$\mathcal{F}_x\left[e^{-ax^2}\right](k) = \sqrt{\frac{\pi}{a}}\, e^{-\pi^2 k^2/a^2}$	The Fourier transform of a Gaussian is a Gaussian.	
10	$e = \lim_{n\to\infty}\left(1 + \frac{1}{n}\right)^n$	An identity for Euler's number *e*.	

#	Equation	Description	Rating
11	$2^{\|S\|} > \|S\|$	The cardinality of the continuum has the same number of elements as the power set of the integers and is greater that the cardinality of the integers.	
12	$z_{n+1} = z_n^2 + c$	Defines the Mandelbrot set, that is a complex number *c* is part of the Mandelbrot set if, when starting with $z_0 = 0$ and applying the iteration repeatedly, the absolute value of z_n remains bounded however large *n* gets.	
13	$f(x) = \int_{-\infty}^{\infty} \delta(x-y)\, f(y) dy$	Identity making use of the Dirac delta function.	
14	$\frac{1}{\pi} = \frac{2\sqrt{2}}{9801} \sum_{k=0}^{\infty} \frac{(4k)!\,(1103 + 26390k)}{(k!)^4\, 396^{4k}}$	Equation expressing the inverse value of π as an infinite sum.	
15	$1729 = 1^3 + 12^3 = 9^3 + 10^3$	The smallest number expressible as the sum of two cubes in two different ways.	
16	$a^2 + b^2 = c^2$	Pythagoras' theorem: In any right-angled triangle, the area of the square whose side is the hypotenuse (the side opposite the right angle) is equal to the sum of the areas of the squares whose sides are the two legs (the two sides that meet at a right angle).	
17	$\frac{d}{dx} \int_a^x f(s)\, ds = f(x)$	The first fundamental theorem of calculus guarantees the existence of antiderivatives for continuous functions.	
18	$\oint_\gamma f(z) dz = 2\pi i \sum \mathrm{Res}(f, a_k)$	Cauchy's residue theorem is a powerful tool to evaluate line integrals of analytic functions over closed curves.	
19	$\frac{dx}{dt} = x(\alpha - \beta y), \qquad \frac{dy}{dt} = -y(\gamma - \delta x)$	Lotka-Volterra equations (predator-prey equations), are a pair of first-order, non-linear, differential equations used to describe the dynamics of biological systems in which two species interact, one a predator and one its prey.	
20	$\frac{\partial \rho}{\partial t} = D \frac{\partial^2 \rho}{\partial x^2}$	Diffusion equation, a partial differential equation which describes density dynamics in a material undergoing diffusion.	

#	Equation	Description	Rating
21	$\pi = \frac{c}{d}$	π is the ratio of a circle's circumference with its diameter.	
22	$\frac{d}{dx}e^x = e^x$	The derivative of an exponential is an exponential.	
23	$f(x) = \sum_{n=0}^{\infty} \frac{f^{(n)}(0)}{n!} x^n$	Taylor expansion of an analytic function.	
24	$A\mathrm{x} = \lambda \mathrm{x}$	Eigenvalue equation for an operator *A*.	
25	$\lVert x + y \rVert \leq \lVert x \rVert + \lVert y \rVert$	Triangle inequality for a normed vector space, the norm of the sum of two vectors is at most as large as the sum of the norms of the two vectors.	
26	$\pi(x) \sim \frac{x}{\log x}$	Prime number theorem: If x is any positive real number, then π(x), which is the number of primes less than x, is approximately x/log x.	
27	$\sum_{n=1}^{\infty} \frac{1}{n^s} = \prod_{p} \left(1 - \frac{1}{p^s}\right)^{-1}$	The Euler product formula for the Riemann zeta function where the left hand side equals the Riemann zeta function and the product on the right hand side extends over all prime numbers *p*.	
28	$3^2 + 4^2 = 5^2$	Pythagoras' theorem for a 3:4:5 triangle.	
29	$\frac{d^n}{dz^n} f(z) = \frac{n!}{2\pi i} \oint_c \frac{f(w)}{(w - z)^{n+1}} dw$	Cauchy's integral formula.	
30	$\frac{\pi}{4} = 1 - \frac{1}{3} + \frac{1}{5} - \frac{1}{7} + \frac{1}{9} - \cdots$	Equation expressing the connection between π and odd numbers.	

#	Equation	Description	Rating
31	$\frac{\pi^2}{6} = \sum_{n=1}^{\infty} \frac{1}{n^2}$	The Basel problem, set as a challenge by Jakob Bernoulli in 1689 and triumphantly solved by Euler in 1735.	
32	$\sum_{k=0}^{\infty} ar^k = \frac{a}{1-r}, \qquad \lvert r \rvert < 1$	Sum of an infinite geometric series	
33	$\int f d\mu = \lim_{n\to\infty} \frac{1}{n+1} \sum_{k=0}^{n} f \circ T^k$	Birkhoff's ergodic theorem.	
34	$\int_{\partial M} \omega = \int_M d\omega$	Stoke's theorem states that the integral of a differential form *ω* over the boundary of some orientable manifold *M* is equal to the integral of its exterior derivative *dω* over the whole of *M*	
35	$\sqrt{\pi} \sum_{n=-\infty}^{\infty} e^{-n^2} = \sum_{n=-\infty}^{\infty} e^{-\frac{n^2}{4}}$	A special case of Poisson summation.	
36	$\langle B, B \rangle_t = t$	The quadratic variation of a one dimensional Brownian motion, up to time t is t. This is basic to both Feynman's path integral approach to quantum mechanics and to stochastic analysis.	
37	$\prod_{k=0}^{\infty} (1 + x^k) = \sum_{n=0}^{\infty} p(n) x^n$	Another equation due to Euler: on the left hand side we have an infinite product and on the right hand side appears a power series where *p(n)* denotes the number of all possible representations of n as a sum of natural numbers.	
38	$\frac{1}{n} \sum_{k=1}^{n} a_k \geq \left(\prod_{k=1}^{n} a_k \right)^{\frac{1}{n}}, \qquad a_k > 0$	An inequality due to Cauchy expressing a relation between a product and a sum.	
39	$\lvert \emptyset \rvert = 0$	The cardinality of the empty set is zero.	
40	$T = de + \omega \wedge e, \qquad R = d\omega + \omega \wedge \omega$	Cartan structural equations, where e is the frame field, omega the connection form, T the torsion form and R the curvature form.	

#	Equation	Description	Rating
41	$n! = n^n e^{-n}\sqrt{2\pi n}\big(1 + o(n)\big)$	Stirling's approximation to n!.	
42	$\chi_\Omega(\exp X) = \int_\Omega e^{i\langle F,X\rangle + \sigma(F)}$	Integral formula for a character of an irreducible representation of a Lie group corresponding to the co-adjoint orbit Ω.	
43	$V_n(r) = \frac{\pi^{\frac{n}{2}}}{\Gamma\left(\frac{n}{2}+1\right)} r^n$	Volume of an n-dimensional sphere.	
44	$S^2 \cong CP^1 \cong SO(3)\ /\ SO(2)$	Relation between the sphere, the complex projective line and the special orthogonal groups SO(3) and SO(2).	
45	$\mathbb{Z} \overset{2.}{\hookrightarrow} \mathbb{Z} \twoheadrightarrow \mathbb{Z}/2\mathbb{Z}$	An example of an exact sequence where the image of one morphism equals the kernel of the next.	
46	$R^{\alpha}_{\beta[\gamma\delta;\lambda]} = 0$	Second Bianchi identity of the Riemann tensor.	
47	$f(z) = \frac{az+b}{cz+d}$	Definition of a Möbius transformation.	
48	$\{\gamma_i, \gamma_j\} = 2\eta_{ij}$	Clifford algebra.	
49	$1 = \sum_{n=2}^{\infty} (\zeta(n) - 1)$	The integer 1 expressed as an infinite series involving the Riemann zeta function	
50	$A \cap A^C = \emptyset$	The intersection of a set with its complement is empty.	

#	Equation	Description	Rating
51	$U^C = \emptyset$	The complement of the universal set is the empty set.	
52	$A = \int_{\sigma(A)} \lambda dE_\lambda$	A formulation of the spectral theorem expresses the operator as an integral of the coordinate function over the operator's spectrum with respect to a projection-valued measure.	
53	$\int \theta d\theta = 1, \qquad \int d\theta = 0$	Properties defining Berezin integration, where theta is an anticommuting variable over the complex numbers.	
54	$\frac{\partial u}{\partial x} = \frac{\partial v}{\partial y}, \qquad \frac{\partial u}{\partial y} = -\frac{\partial v}{\partial x}$	Cauchy-Riemann equations are a system of two partial differential equations which must be satisfied if a complex function is complex differentiable.	
55	$\Delta\varphi = 0$	Laplace equation.	
56	$x^2 - ny^2 = 1$	Pell's diophantine equation.	
57	$\varphi_{tt} - \varphi_{xx} + \sin\varphi = 0$	Sine-Gordon equation, a nonlinear hyperbolic partial differential equation in 1 + 1 dimensions.	
58	$a^n + b^n = c^n, \qquad n > 2$	Fermat's Last Theorem states that no three positive integers a, b, and c can satisfy this equation for any integer value of n greater than two.	
59	$\zeta(s) = 2^s \pi^{s-1} \sin\left(\frac{\pi s}{2}\right) \Gamma(1-s) \zeta(1-s)$	Riemann's functional equation, where Γ(s) is the gamma function and ζ(s) is the Riemann zeta-function	
60	$\nabla_\mu \left(R^{\mu\nu} - \frac{1}{2} g^{\mu\nu} R\right) = 0$	Contracted Bianchi identity where $R^{\mu\nu}$ is the Ricci tensor and *R* is the scalar curvature	
