# Supplementary material for: The experience of mathematical beauty and its neural correlates
Source: Front Hum Neurosci. 2014 Feb 13;8:68. doi: 10.3389/fnhum.2014.00068 (PMC3923150; doi:10.3389/fnhum.2014.00068)
Supplement: Data Sheet 4 [file DataSheet4.PDF]

Activations - *Beautiful* > *Baseline*

x	y	z	k_E	T_{14}	P_{FWE}
**-36**	**-82**	**-8**	**181**	**14.90**	**< 0.001**
-30	-88	-8		14.79	< 0.001
**42**	**-79**	**-11**	**300**	**13.43**	**< 0.001**
45	-67	-29		10.57	0.001
27	-97	1		10.06	0.002
**-48**	**47**	**4**	**76**	**11.73**	**< 0.001**
**-48**	**-61**	**-11**	**97**	**10.83**	**0.001**
-48	-46	-8		8.86	0.012
-36	-67	-20		8.06	0.034
**30**	**-55**	**49**	**21**	**9.49**	**0.005**
**39**	**-58**	**-14**	**25**	**9.41**	**0.006**
48	-52	-14		7.89	0.041
**-30**	**-67**	**46**	**13**	**8.32**	**0.025**

De-activations - *Beautiful* < *Baseline*

x	y	z	k_E	T_{14}	P_{FWE}
**-15**	**-70**	**-2**	**29**	**14.99**	**< 0.001**
**57**	**-16**	**-8**	**59**	**14.39**	**< 0.001**
**60**	**5**	**-23**	**70**	**13.65**	**< 0.001**
57	-1	-11		8.68	0.015
**3**	**32**	**-2**	**164**	**12.95**	**< 0.001**
-6	23	-11		11.06	0.001
9	53	1		10.93	0.001
**3**	**-55**	**25**	**719**	**12.77**	**< 0.001**
12	-73	28		12.67	< 0.001
-12	-43	43		11.88	< 0.001
**60**	**-52**	**16**	**170**	**11.46**	**< 0.001**
63	-34	16		9.91	0.003
57	-37	4		9.10	0.009
**-57**	**-4**	**-17**	**32**	**10.58**	**0.001**
**18**	**50**	**19**	**12**	**10.29**	**0.002**
**3**	**-88**	**16**	**11**	**8.55**	**0.018**
3	-85	10		7.86	0.042
**42**	**-13**	**-8**	**15**	**8.51**	**0.019**

Activations - *Neutral* > *Baseline*

x	y	z	k_E	T_{14}	P_{FWE}
**-30**	**-91**	**-5**	**202**	**15.09**	**< 0.001**
-33	-82	-8		14.44	< 0.001
**42**	**-79**	**-11**	**361**	**14.75**	**< 0.001**
45	-49	-11		12.02	< 0.001
45	-73	-26		11.90	< 0.001
**-48**	**47**	**4**	**38**	**13.54**	**< 0.001**
**-27**	**-67**	**55**	**121**	**11.40**	**< 0.001**
-30	-52	55		10.97	0.001
-27	-67	37		8.97	0.010
**-48**	**-61**	**-11**	**67**	**11.27**	**0.001**
**30**	**-70**	**-50**	**14**	**10.98**	**0.001**
**6**	**-79**	**-29**	**104**	**10.03**	**0.001**
-9	-76	-26		9.96	0.003
12	-76	-41		8.70	0.014
**-45**	**5**	**37**	**36**	**9.94**	**0.003**
**-6**	**20**	**43**	**10**	**9.53**	**0.005**
**27**	**-58**	**40**	**23**	**9.43**	**0.005**
30	-52	46		8.17	0.031

De-activations - *Neutral* < *Baseline*

x	y	z	k_E	T_{14}	P_{FWE}
**-12**	**-46**	**43**	**634**	**14.58**	**< 0.001**
-6	-49	25		12.77	< 0.001
0	-55	28		12.75	< 0.001
**60**	**-52**	**16**	**88**	**14.11**	**< 0.001**
**-9**	**47**	**-5**	**585**	**13.40**	**< 0.001**
-9	38	-2		12.92	< 0.001
12	53	-2		12.71	< 0.001
**-63**	**-4**	**-17**	**31**	**11.93**	**< 0.001**
**57**	**-19**	**-8**	**101**	**10.74**	**0.001**
54	-7	-20		10.50	0.001
60	-1	-20		10.47	0.002
**9**	**-91**	**16**	**16**	**9.40**	**0.006**

Activations and de-activations for the categorical contrasts *Beautiful and Neutral vs Baseline*. SPM 2nd level analyses (15 subjects). One sample t-test (df = 14). All loci, in Montreal Neurological Institute (MNI) space, are significant at peak level, thresholded at $\mathbf{P_{FWE}} < 0.05$ with familywise error correction over the whole brain volume and with an extent threshold of 10 voxels. The peak activation in each cluster is shown in bold with the cluster size in voxels ($\mathbf{k_E}$) with up to two other peaks in the same cluster listed below.

Activations - *Ugly* > *Baseline*

x	y	z	k_E	T_{14}	P_{FWE}
**-36**	**-79**	**-8**	**266**	**15.36**	**< 0.001**
-30	-91	-5		15.26	< 0.001
-48	-61	-8		10.28	0.002
**39**	**-85**	**-8**	**280**	**14.45**	**< 0.001**
42	-61	-11		11.07	0.001
27	-97	1		10.70	0.001
**-24**	**-67**	**55**	**37**	**11.23**	**0.001**
-27	-49	49		10.98	0.001

De-activations - *Ugly* < *Baseline*

x	y	z	k_E	T_{14}	P_{FWE}
**63**	**-46**	**16**	**165**	**14.87**	**< 0.001**
54	-37	22		9.36	0.006
**6**	**35**	**-5**	**249**	**14.85**	**< 0.001**
-6	35	-2-		13.08	< 0.001
-12	44	-2		11.49	< 0.001
**60**	**-19**	**-2**	**255**	**14.25**	**< 0.001**
54	-4	-20		13.25	< 0.001
48	-16	-5		13.00	< 0.001
**-12**	**-40**	**49**	**99**	**12.86**	**< 0.001**
-12	-46	43		10.65	0.001
**12**	**-40**	**43**	**68**	**12.26**	**< 0.001**
6	-25	40		8.87	0.012
**12**	**-73**	**31**	**110**	**10.39**	**0.002**
-9	-67	25		9.86	0.003
-15	-52	25		9.80	0.003
**-57**	**-4**	**-17**	**16**	**9.76**	**0.004**
**15**	**-49**	**25**	**10**	**9.41**	**0.006**

Activations and de-activations for the categorical contrast *Ugly vs Baseline*. SPM 2nd level analyses (15 subjects). One sample t-test (df = 14). All loci, in Montreal Neurological Institute (MNI) space, are significant at peak level, thresholded at $\mathbf{P_{FWE}} < 0.05$ with familywise error correction over the whole brain volume and with an extent threshold of 10 voxels. The peak activation in each cluster is shown in bold with the cluster size in voxels ($\mathbf{k_E}$) with up to two other peaks in the same cluster listed below.
